# Spatial genomic heterogeneity in diffuse intrinsic pontine and midline high-grade glioma: implications for diagnostic biopsy and targeted therapeutics

**DOI:** 10.1186/s40478-015-0269-0

**Published:** 2016-01-04

**Authors:** Lindsey M. Hoffman, Mariko DeWire, Scott Ryall, Pawel Buczkowicz, James Leach, Lili Miles, Arun Ramani, Michael Brudno, Shiva Senthil Kumar, Rachid Drissi, Phillip Dexheimer, Ralph Salloum, Lionel Chow, Trent Hummel, Charles Stevenson, Q. Richard Lu, Blaise Jones, David Witte, Bruce Aronow, Cynthia E. Hawkins, Maryam Fouladi

**Affiliations:** Cincinnati Children’s Hospital Medical Center, 3333 Burnet Avenue, Cincinnati, OH 45229 USA; The Hospital for Sick Children, Toronto, Canada

**Keywords:** Genomic heterogeneity, Pediatric, Glioma, Targeted therapeutics

## Abstract

**Introduction:**

Diffuse intrinsic pontine glioma (DIPG) and midline high-grade glioma (mHGG) are lethal childhood brain tumors. Spatial genomic heterogeneity has been well-described in adult HGG but has not been comprehensively characterized in pediatric HGG. We performed whole exome sequencing on 38-matched primary, contiguous, and metastatic tumor sites from eight children with DIPG (*n* = 7) or mHGG (*n* = 1) collected using a unique MRI-guided autopsy protocol. Validation was performed using Sanger sequencing, Droplet Digital polymerase-chain reaction, immunohistochemistry, and fluorescent in-situ hybridization.

**Results:**

Median age at diagnosis was 6.1 years (range: 2.9–23.3 years). Median overall survival was 13.2 months (range: 11.2–32.2 months). Contiguous tumor infiltration and distant metastases were observed in seven and six patients, respectively, including leptomeningeal dissemination in three DIPGs. Histopathological heterogeneity was evident in seven patients, including intra-pontine heterogeneity in two DIPGs, ranging from World Health Organization grade II to IV astrocytoma. We found conservation of heterozygous K27M mutations in H3F3A (*n* = 4) or HIST1H3B (*n* = 3) across all primary, contiguous, and metastatic tumor sites in all DIPGs. ACVR1 (*n* = 2), PIK3CA (*n* = 2), FGFR1 (*n* = 2), and MET (*n* = 1) were also intra-tumorally conserved. ACVR1 was co-mutated with HIST1H3B (*n* = 2). In contrast, PDGFRA amplification and mutation were spatially heterogeneous, as were mutations in BCOR (*n* = 1), ATRX (*n* = 2), and MYC (*n* = 1). TP53 aberrations (*n* = 3 patients) varied by type and location between primary and metastatic tumors sites but were intra-tumorally conserved.

**Conclusion:**

Spatial conservation of prognostically-relevant and therapeutically-targetable somatic mutations in DIPG and mHGG contrasts the significant heterogeneity of driver mutations seen in adult HGG and supports uniform implementation of diagnostic biopsy in DIPG and mHGG to classify molecular risk groups and guide therapeutic strategy.

**Electronic supplementary material:**

The online version of this article (doi:10.1186/s40478-015-0269-0) contains supplementary material, which is available to authorized users.

## Introduction

Diffuse intrinsic pontine glioma (DIPG) is an aggressive pediatric brain tumor with a median survival of less than 1 year, despite current multimodal therapies [1, 2]. Midline, non-brainstem high-grade gliomas (mHGGs) in children share clinical and biological features with DIPG and have a similarly dismal prognosis [3–5]. Historically, reluctance to biopsy these precariously located tumors to obtain tissue has impeded the understanding of their biology. Recently, greater acceptance of the safety of biopsy [6–9], development of autopsy-based protocols [10–12], and advancement of high-throughput sequencing technology have enabled unprecedented insight into the molecular underpinnings of DIPG and mHGG. Genomic studies have detailed recurrent aberrations in many canonical cancer pathways and mutations in novel oncogenes, such as highly recurrent histone mutations (*H3F3A* or *HIST1H3B/C/I*) and *ACVR1* [5, 10, 13, 14].

Despite remarkable genomic discoveries, therapeutic progress for DIPG and mHGG has remained static. The standard treatment, focal radiotherapy, provides only transient local control and fails to address the recently reported metastatic potential of these highly infiltrative tumors [1, 15, 16]. Clinical trials of adjuvant chemotherapy and targeted therapy, including those targeting critical pathways, such as platelet-derived growth factor receptor alpha (PDGFRA) [7–9, 17], have not improved outcome over many decades [11, 12, 18]. Intra-tumoral genomic heterogeneity and clonal evolution are well-recognized in the pathogenesis and therapeutic resistance of adult HGG [19–21]. We previously reported intra-tumoral histopathological variation in DIPGs from autopsy [1]. However, evaluation of spatial genomic heterogeneity, which carries important implications for determining the generalizability of molecular profiles derived from small diagnostic biopsies and scientifically-sound integration of molecularly-targeted therapies, has not been reported in DIPG or mHGG. We used whole exome sequencing (WES) to comprehensively evaluate spatial intra-tumoral genomic heterogeneity in eight children with DIPG or mHGG.

## Materials and methods

### Patient cohort

Tissue samples (*n* = 38) and clinical data were obtained from eight patients with DIPG (*n* = 7) or mHGG (*n* = 1) consented to the IRB-approved Pediatric Brain Tumor Repository (PBTR) autopsy protocol (Study: 2013–1245) at Cincinnati Children’s Hospital Medical Center from 2013–2014. Diagnosis of DIPG was based on rapidly progressive clinical symptoms and imaging characteristics on pre-treatment magnetic resonance imaging (MRI) (infiltrative tumor involving at least two-thirds of the pons). One patient with a bi-thalamic tumor underwent biopsy (not sequenced); no DIPGs were biopsied. Diagnostic and post-mortem imaging for all patients was centrally reviewed by one neuro-radiologist (J.L.).

#### Autopsy protocol

**Fig. 1:**
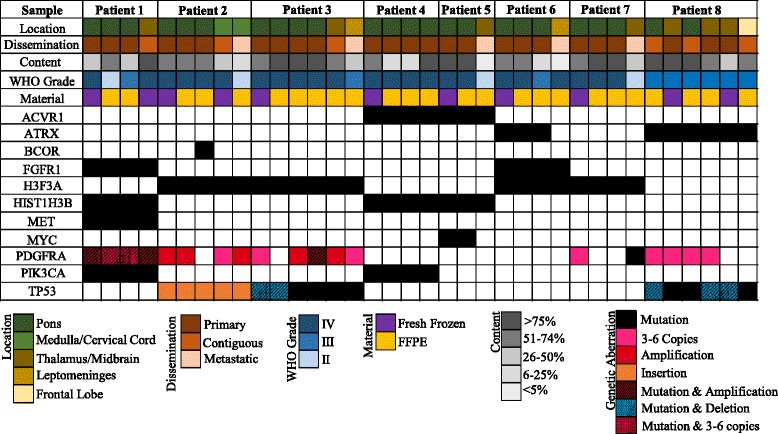
Spatial histopathological and genomic landscape of DIPG and mHGG in children. Histology and WHO grade were frequently heterogeneous within the pons or between the pons and contiguous or metastatic sites. Somatic mutations (indicated by a black box or stripped overlay of a colored box if co-existing with a copy number change) in *H3F3A* (H3.3), *HIST1H3B* (H3.1), *ACVR1*, *PIK3CA*, *FGFR1*, and *MET* were conserved across all disease sites. Somatic mutations in *ATRX, BCOR*, *MYC*, and *PDGFRA* and *PDGFRA* amplification were spatially heterogeneous. WHO = World Health Organization, FFPE = formalin-fixed paraffin-embedded

Median time from death to autopsy was 11.8 h (range: 4.8–20 h). Time to autopsy did not affect the quality or quantity of DNA. Normal brain and tumor visible on gross examination, including at least two locations in the pons (usually left anterior and right posterior), were sampled when the brain was harvested from the calvarium. After harvest, the whole brain was fixed in formalin. *Ex-vivo* MRI was performed on the fixed whole brain of seven patients (Patient 5 not imaged due to poor anatomical configuration after harvest) using a 1.5-Tesla MR system employing high-resolution volumetric T1- and T2-weighted images. 3D renderings were performed to optimize orientation of gradient-echo T1. Coronal and axial oblique images were obtained on the cerebrum and cerebellum, respectively. Volumetric data sets were reconstructed into standard anatomic orientations, and all attempts were made to duplicate standard in vivo slice orientation for planar data sets. The pathologist (L.M.) and neuro-radiologist jointly reviewed hematoxylin & eosin (H&E) sections and MRI images to determine contiguous and metastatic tumor locations. Tumor was classified as “primary” if located within the pons (or thalamus in Patient 8), “contiguous” if involving a CNS structure adjoining the primary tumor, or “metastatic” if involving a non-contiguous CNS structure. Routine sections of the frontal lobe, cerebellar cortex, all deep gray nuclei of cerebrum/cerebellum, and all brainstem structures were taken regardless of gross and/or MRI abnormalities. Two neuropathologists (L.M., C.H.) independently reviewed each tumor sample to assign histology, grade, and tumor percentage. To ensure adequate tumor content (Fig. 1), H&E slides were reviewed from each frozen specimen, the initial cut of each formalin-fixed paraffin-embedded (FFPE) block, and an additional cut of FFPE block after scrolls were obtained for DNA extraction.

### Whole exome sequencing alignment and structural variants

DNA extraction was performed on frozen tissue as previously described [22] and on FFPE tissue using Biochain’s FFPE Tissue DNA Extraction Kit (#K5019100). Library preparation and WES were performed at BGI Americas using the Agilent SureSelect Human All Exon V5 Kit and Illumina HiSeq 2500 using V3 chemistry, respectively. All samples were sequenced at an average depth of 100× (4 samples per lane generating 12 Gb of raw data per sample). The Burrows-Wheeler Aligner [23] tool was used to map all reads to the human genome build GRCh37/hg19. The GATK variant calling pipeline was followed to identify single nucleotide polymorphisms. MuTect 1.1.4 was used to detect somatic single nucleotide variants (SNVs) in 46 candidate genes with clinical significance (Additional file 1: Table S1). Variants with sufficient coverage were further annotated using Annovar and SNPeff, giving RefSeq gene annotations, amino acid changes, ExAC SIFT, PolyPhen, LTR and MutationTaster scores.

### Histology and immunohistochemistry

H&E staining and p53 immunohistochemistry (IHC) [p53 (DO-7) primary mouse antibody 790–2912] were performed on 4 μm paraffin sections mounted on positively charged slides. Immuno-detection was performed as previously described [1].

### Sanger sequencing and droplet digital polymerase-chain reaction

*H3F3A* and *HIST1H3B* were partially sequenced as described by Wu et al. [1, 24] (Additional file 2: Table S2). Somatic SNVs identified by WES were validated using Droplet Digital PCR System according to manufacturer instructions (BioRad, Mississauga, Canada) (Additional file 3: Table S3). Samples were read in duplicate, and data were considered if at least 100 droplets contained fluorescent signal from either VIC or FAM channels in both duplicates. Samples were considered positive for mutation if at least 10 positive droplets were detected in the mutant channel (FAM). Mutational allele frequencies were estimated using a ratio between the mutant channel signal and total positive droplet counts.

### Florescence in-situ hybridization

Florescence in-situ hybridization (FISH) for PDGFRA (Empire Genomics dual-color break apart probe) and c-MYC (Abbott Molecular dual-color break apart probe) was performed on 4 μm paraffin sections mounted on positively charged slides with DAPI II counterstain.

### Statistics

Bioinformatic methods are described above. Descriptive statistics were used to report the frequency of genetic changes across disease locations.

## Results

### Clinical and histopathological characteristics

Patient characteristics and treatment data are listed in Table 1. Median age at diagnosis was 6.1 years (range: 2.9-23.3 years); median overall survival (OS) was 13.2 months (range: 11.2–32.2 months). Median time between the last pre- and post-mortem imaging was 46 days (range: 28–448 days). Only Patient 1 had evidence of distant metastases on pre-mortem imaging (5 months after diagnosis). Primary tumors were glioblastoma (GBM, *n* = 7) or anaplastic astrocytoma (AA, *n* = 1) (Fig. 1). Intra-tumoral histopathological heterogeneity was observed in the primary tumor of seven of eight patients, including the primary tumor in Patient 1 that demonstrated regions with WHO grade II, III, and IV astrocytoma. The histological grade of primary and metastatic sites varied in four of five DIPG patients with metastatic disease. Intracranial leptomeningeal dissemination was observed in three of seven DIPGs (leptomeningeal disease for Patient 1 was not sequenced) (Fig. 1).

**Table 1:** Clinical characteristics of patients with DIPG and mHGG

Patient	Age at Diagnosis (years)	Sex	Primary Tumor	Treatment at Diagnosis	OS (months)	Time to Autopsy (hours)
1	7.7	Male	DIPG	XRT + bevacizumab, irinotecan	24.4	19
2	2.9	Male	DIPG	XRT	32.2	17.5
3	4.3	Male	DIPG	XRT + HDAC inhibitor	11.2	15.5
4	4.4	Male	DIPG	XRT + HDAC inhibitor	11.5	4.8
5	3.5	Male	DIPG	XRT + EGFR inhibitor	16.8	5.5
6	23.3	Male	DIPG	XRT + HDAC inhibitor	13.4	8
7	12.3	Female	DIPG	XRT + PARP inhibitor, TMZ	11.8	20
8	15.1	Female	Bi-thalamic HGG	XRT + bevacizumab, TMZ, irinotecan	13.2	8

*DIPG* diffuse intrinsic pontine glioma, *HGG* high-grade glioma, *TMZ* temozolomide, *HDAC* histone deacetylase, *PARP* poly ADP ribose polymerase, *EGFR* epidermal growth factor receptor, *OS* overall survival, *XRT* radiotherapy

### Lack of heterogeneity in core gene set supports their role as driver mutations and potential therapeutic targets

Relevant genomic findings are summarized in Fig. 1. Heterozygous H3K27M mutations were detected by WES across all primary, contiguous, and metastatic disease sites in all seven DIPG patients and were validated by ddPCR (Additional file 4: Table S4) and Sanger sequencing (Additional file 5: Figure S1). H3.3-K27M was found in four DIPGs and co-occurred with *PDGFRA* amplification and *TP53* aberrations in two cases, an association we previously described [14, 22]. *ACVR1* mutation (G328V/W) was found in two patients, both with H3.1-K27M. Mutations in *ACVR1* (2 patients), *FGFR1* (K697E or N98S) (two patients), *MET* (S572N) (1 patient), and *PIK3CA* (E545K or I291M) (2 patients) were conserved across all disease locations, as confirmed by ddPCR (Additional file 4: Table S4). *TP53* aberrations were observed in three patients, including deletion and/or mutation, across all disease sites in Patient 8 (Additional file 6: Figure S2), who did not harbor a histone mutation, suggesting *TP53* as a potential driver. Varied *TP53* aberrations were also found across all disease locations in two DIPGs with H3.3-K27M mutations, perhaps representing a secondary but important genetic event.

### Gene mutations demonstrating spatial heterogeneity suggest secondary hits and poor potential for monotherapy

**Fig. 2:**
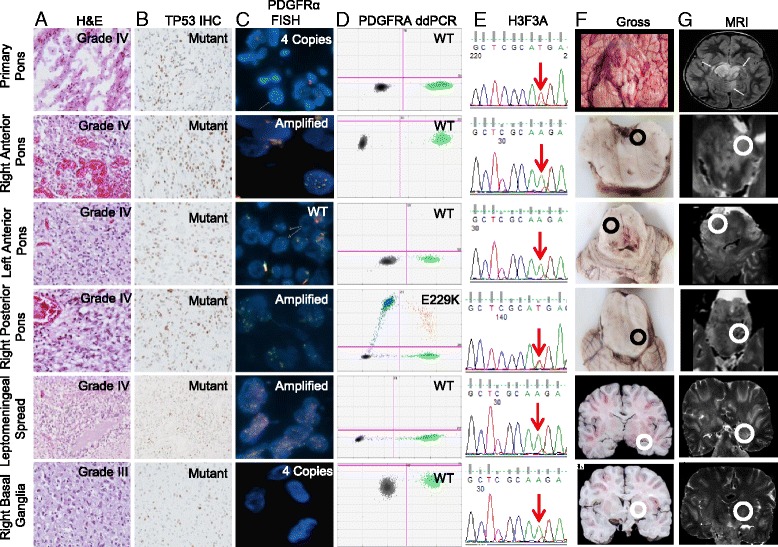
Intra-tumoral heterogeneity in a 4-year old male with DIPG (Patient 3). Each row represents a distinct disease location, including four different areas in the primary tumor, one contiguous lesion in the right basal ganglia, and one metastatic leptomeningeal lesion. **a** H&Es of each tumor location demonstrate WHO grade IV histology in all except the right basal ganglia lesion (WHO grade III), **b** P53 IHC demonstrates variable positivity, **c** *PDGFRA* FISH demonstrates gain or amplification in 3 of 4 sites within the pontine tumor, gain in the contiguous right basal ganglia lesion, and amplification in the metastatic leptomeningeal lesion, **d** *PDGFRA* ddPCR demonstrating PDGFRA mutation observed in the right posterior pons, **e** *H3F3A* was mutated in all samples as demonstrated by Sanger sequencing, **f** sample location taken from fresh (“Primary Pons”, *Row 1*) or fixed tissue (*remaining rows*) at autopsy corresponding to tumor location identified on post-mortem MRI imaging (**g**). Because post-mortem imaging was only performed on fixed tissue, MRI imaging for the fresh tissue sample (“Primary Pons”, *Row 1*) was a pre-mortem MRI performed approximately 6 weeks prior to death

*PDGFRA* point mutations occurred in two of eight patients. All disease sites in Patient 1 harbored two *PDGFRA* mutations (S247L, Y555C), but only 1 of 6 disease sites in Patient 3, which also exhibited high-level *PDGFRA* amplification, harbored a *PDGFRA* mutation (E229K) (Fig. 2). *PDGFRA* gain/amplification was observed in five patients and showed notable intra-tumoral heterogeneity (Additional file 7: Figure S3), suggesting clonality of this recurrent genetic event. Other intra-tumorally heterogeneous mutations included *ATRX*, *BCOR*, and *MYC*. Heterozygous *ATRX* mutations were found in Patients 6 (H2254R) and 8 (R2197L); while conserved across all disease sites in Patient 8, the *ATRX* mutation in Patient 6 was absent in the metastatic compartment. A heterozygous *BCOR* mutation (A535V) was found in a single pontine site in Patient 2, and a heterozygous *MYC* mutation (Q51L) was found in both pontine sites in Patient 5 but was absent in the metastatic lesion. FISH confirmed no *MYC* copy number gain in any tumor samples from Patient 5.

Except for *TP53*, we did not detect somatic mutations in genes involved in cell-cycle regulation, such as *CDK4/6*, *CCND1/2/3*, *CDKN2A/B* or *RB1*. Amplification of *CDK4/6* or *CCND1/2/3*, more common in DIPG [14], was not assessed. As expected, H3G34R/V mutations, predominantly reported in hemispheric pediatric HGGs [25], were also not found.

## Discussion

Recent large-scale genomic studies have established the inter-patient molecular complexity of pediatric HGGs [5, 10, 13, 14, 24]; however, limited understanding of spatial subclonal architecture has impeded development of effective therapies. To our knowledge, we are the first to report multi-regional genetic analyses of matched primary, contiguous, and metastatic sites from DIPG and mHGG. Disease-defining somatic mutations in *H3F3A*, *HIST1H3B*, and *ACVR1* were conserved across all tumor locations, as were mutations affecting the (RTK)-PI3K-MAPK pathway. However, other targetable molecular aberrations, such as *PDGFRA*, were spatially heterogeneous. Findings from this study reiterate the distinct biology between pediatric and adult HGG and are critical for establishing the role of diagnostic biopsy and informing therapeutic strategy for these lethal pediatric tumors.

Our findings confirm the propensity of DIPG and mHGG for aggressive local and distant metastatic spread (Fig. 1) and are consistent with our prior study in which extra-brainstem and leptomeningeal disease was found in 25 and 39 % of DIPGs from autopsy, respectively [1]. Caretti et al. similarly reported leptomeningeal involvement in a quarter of DIPGs from autopsy [26]. Importantly, we found histological evidence of metastatic disease by post-mortem MRI in five cases in which tumor was not seen on pre-mortem imaging or grossly at autopsy. Hence, disease dissemination is prevalent, but in most cases appears late in the disease course.

Spatial histological heterogeneity, frequently reported in adult GBM [27, 28], was observed in all but one patient in our cohort (Fig. 1). Tissue from two DIPGs demonstrated intra-pontine histologic variation, including one with evidence of WHO grade II, III, and IV astrocytoma. We previously reported substantial histologic heterogeneity at various levels of the brainstem in DIPGs from autopsy [1]; other autopsy studies have reported limited histologic evaluation of one pontine site, most of which were high-grade [12]. Metastatic lesions in our cohort tended to be of lower histologic grade, a finding consistent with our prior report [1]. While variation in metastatic regions may represent sampling bias in areas of lower tumor content, our finding of histologic heterogeneity within the densely tumor-packed pons in DIPG reiterates the poor reliability of histopathological grading for clinical stratification [1].

Significant temporal and spatial genetic and epigenetic heterogeneity has been reported in adult HGG, including regional variation of known driver mutations *EGFR*, *MET*, *PTEN*, and *CDK6* [19], as well as heterogeneity of *MGMT* promoter methylation [29]. We are the first to undertake comprehensive evaluation of the spatial genomic landscape of DIPG and mHGG in children. The most significant genomic discovery in DIPG and mHGG, to date, has been that of recurrent mutations in evolutionarily highly-conserved histone genes *H3F3A* and *HIST1H3B*, resulting in replacement of lysine 27 by methionine (K27M); these mutually exclusive somatic alterations occur in approximately 80 % of DIPGs [14] and 50 % of pediatric thalamic GBMs [4, 5]. Unlike the remarkable intra-tumoral heterogeneity of driver mutations in adult HGG, we confirmed the presence of heterozygous K27M mutations in *H3F3A* or *HIST1H3B* in 100 % of tumor cells across all disease compartments in four and three DIPGs, respectively. This finding builds on prior reports demonstrating consistently high allelic frequency of histone mutations in all primary DIPG tumor cells using deep sequencing [1, 30] and Kambhampati et al. finding of conservation of H3.3-K27M by IHC between the pons and ventricular tumor extension in one DIPG patient from autopsy [11]. Activating point mutations of *ACVR1*, present in 20–30 % of DIPGs [5, 14] and potentially targetable, were also conserved across all tumor compartments. *ACVR1* mutations were strongly associated with H3.1-K27M, as previously described [14]. Furthermore, spatial conservation of activating mutations of *FGFR1*, *PIK3CA,* and *MET* supports the therapeutic relevance of targeting the (RTK)-PI3K-MAPK pathway in DIPG. Spatial homogeneity of *PIK3CA* mutations, in particular, supports their role as “founder mutations” in pediatric HGG, as suggested by Wu et al. who discovered a common *PIK3CA* mutation (Q546K) in multiple tumor subclones derived from matched mHGG samples from diagnosis and recurrence, suggesting not only spatial but also longitudinal conservation [14]. Although *TP53* aberrations were present across all tumor compartments, the type and location of aberration varied suggesting that they are critical, but likely secondary hits.

Similar to adult HGG, we observed subclonal variation of *PDGFRA* aberrations in our cohort. Spatial heterogeneity was particularly evident for Patient 3 in whom amplification was observed in three of six tumor locations (Fig. 2). Fontebasso et al. similarly reported amplification in one of five primary tumor sites in a treatment-naive mHGG [5]. Interestingly, in Patient 3, one pontine site bearing high-level *PDGFRA* amplification also harbored a missense *PDGFRA* mutation (E229K); other sites with *PDGFRA* gain/amplification were wild-type with sufficient read coverage, suggesting later acquisition of this activating mutation in *PDGFRA*-amplified clones. Our findings are similar to Puget et al. who reported co-occurrence of *PDGFRA* mutation and amplification in three treatment-naïve DIPGs [31] but differ from a report by Paugh et al. who found mutual exclusivity of amplification and mutation in 26 and 5 % of 43 DIPGs, respectively [32]. While it is apparent that *PDGFRA* mutations and copy number changes arise in subclonal populations, further studies are needed to determine their temporal order and functional consequence.

Our description of the spatial genomic landscape of DIPG and mHGG provides critical insight into the utility of diagnostic biopsy and the biologic rationale behind selection of therapeutic targets. Since the 1980s, imaging, rather than tissue, diagnosis has been the standard for DIPG and most mHGGs. More recently, pre-treatment biopsy has gained wider acceptance given the procedure’s low morbidity [7, 8] and discovery of potentially actionable genetic alterations that may inform therapy [5, 6, 14]. Despite a notable shift in perspective in the pediatric neuro-oncology community, ongoing concerns about intrinsic heterogeneity, poor ability to decipher driver from bystander mutations, and sampling bias from the small tissue yield of stereotactic biopsy have impeded uniform implementation of pre-treatment biopsy to guide therapy. Our study offers several important insights in favor of diagnostic biopsy for DIPG and mHGG, including definitive demonstration that disease-driving histone mutations are intra-tumorally conserved. Several large clinico-genomic studies have demonstrated the prognostic relevance of H3 mutations [33, 34]. Ongoing acquisition of pre-treatment tissue will allow refinement of histone mutation-based risk groups and molecular signatures, which have potential to elucidate oncogenic mechanisms and resistance pathways. Our findings also demonstrate that stereotactic biopsy of the safest intracranial disease location offers *bona fide* representation of certain molecular aberrations, abrogating the need for multiple biopsies at different sites of primary or metastatic tumor to elucidate the molecular signature from which therapy may be informed. Furthermore, the finding from this and other reports [1, 32] that primary and metastatic tumor sites may demonstrate variable histopathological grades of astrocytoma (grades II-IV) without affecting prognosis or defining risk groups also supports the recommendation to limit the number of biopsy locations. Stereotactic biopsy of DIPG and mHGG should only be performed at specialized medical centers with skilled pediatric neurosurgeons trained in such techniques to minimize risk.

Development of adjuvant therapies should be focused on targeting highly conserved genetic aberrations, which likely represent true disease drivers. Promising preclinical data demonstrating potent ability of demethylase inhibitors (e.g. GSKJ4) to reverse the broad epigenetic dysregulation and transcriptional signature induced by H3 mutations in DIPG support ongoing efforts for clinical translation of such agents [3, 7, 8]. Grasso et al. also reported compelling in vivo data demonstrating the therapeutic efficacy of multi-histone deacetylase inhibitor panobinostat in H3-mutant DIPG xenografts, including synergistic effect with GSKJ4 [5, 6, 14]. Ongoing work with ALK2 inhibitors for *ACVR1*-mutant DIPG is also encouraging [10, 33, 34]. Therapies directed against subclonal variants in DIPG and mHGG, such as *PDGFRA*, should not be abandoned but rather pursued in combination instead of monotherapy. Therapeutic implications of our findings should be formally tested in clinical trials implementing biopsy-directed targeted therapies. Indeed, target-based stratification based on biopsy results is already underway in several ongoing clinical trials in newly-diagnosed DIPGs (NCT01182350, NCT02233049).

## Conclusion

Though conclusions from our study must be validated in a larger cohort, preferably including diagnostic and autopsy tissue for longitudinal comparison, our findings have immediate clinical relevance for children with DIPG and mHGG by supporting broader implementation of diagnostic biopsy to facilitate ongoing biologic discovery, further define molecular risk groups, and inform therapeutic strategy.

## Additional files

Additional file 1: Table S1. Whole exome sequencing results on 46 candidate genes with previous clinical significance. (XLS 89 kb) Additional file 2: Table S2. H3F3A and HIST1H3B Sanger Sequencing Primers. (XLS 8 kb) Additional file 3: Table S3. ddPCR primer and probe design. (XLS 14 kb) Additional file 4: Table S4. Digital droplet raw droplet counts and mutant allele frequency. (XLS 22 kb) Additional file 5: Figure S1. Chromats of sanger sequencing results for a) HIST1H3B and b) H3F3A K27M. (PDF 245 kb) Additional file 6: Figure S2. Immunohistochemistry validation of TP53 mutants. (PDF 216 kb) Additional file 7: Figure S3. FISH validation for PDGFRA copy number alterations. (PDF 354 kb)
