# Erratum: Spatial genomic heterogeneity in diffuse intrinsic pontine and midline high-grade glioma: implications for diagnostic biopsy and targeted therapeutics

**DOI:** 10.1186/s40478-016-0283-x

**Published:** 2016-02-09

**Authors:** Lindsey M. Hoffman, Mariko DeWire, Scott Ryall, Pawel Buczkowicz, James Leach, Lili Miles, Arun K. Ramani, Michael Brudno, Shiva Senthil Kumar, Rachid Drissi, Phillip Dexheimer, Ralph Salloum, Lionel Chow, Trent Hummel, Charles Stevenson, Q. Richard Lu, Blaise Jones, David Witte, Bruce Aronow, Cynthia E. Hawkins, Maryam Fouladi

**Affiliations:** Cincinnati Children’s Hospital Medical Center, 3333 Burnet Avenue, Cincinnati, OH 45229 USA; The Hospital for Sick Children, Toronto, Canada

Through inadvertent oversight of the authors, the paper [1] failed to acknowledge funding support from Genome Canada. The Acknowledgement section should include the text:

“This work was supported by the Canadian Centre for Computational Genomics (C3G), part of the Genome Innovation Network (GIN), funded by Genome Canada through Genome Quebec and Ontario Genomics”
