# Supplementary material for: Spatial genomic heterogeneity in diffuse intrinsic pontine and midline high-grade glioma: implications for diagnostic biopsy and targeted therapeutics
Source: Acta Neuropathol Commun. 2016 Jan 4;4:1. doi: 10.1186/s40478-015-0269-0 (PMC4700584; doi:10.1186/s40478-015-0269-0)
Supplement: Additional file 5: Figure S1. — Chromats of sanger sequencing results for a) HIST1H3B and b) H3F3A K27M. (PDF 245 kb) [file 40478_2015_269_MOESM5_ESM.pdf]

a.

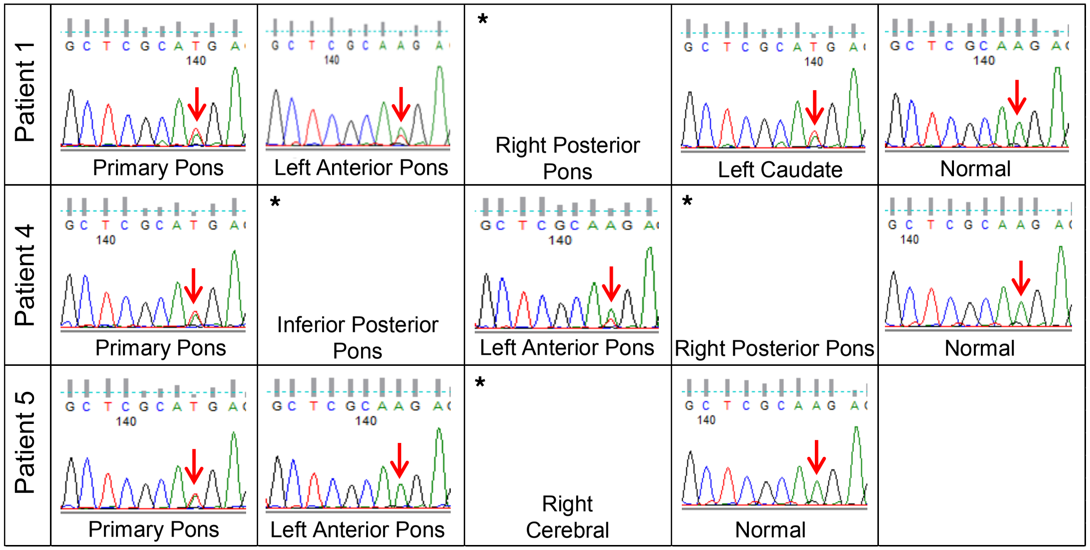

Patient 1
Primary Pons
Left Anterior Pons
*
Right Posterior Pons
Left Caudate
Normal
Patient 4
Primary Pons
*
Inferior Posterior Pons
Left Anterior Pons
*
Right Posterior Pons
Normal
Patient 5
Primary Pons
Left Anterior Pons
*
Right Cerebral
Normal

b.

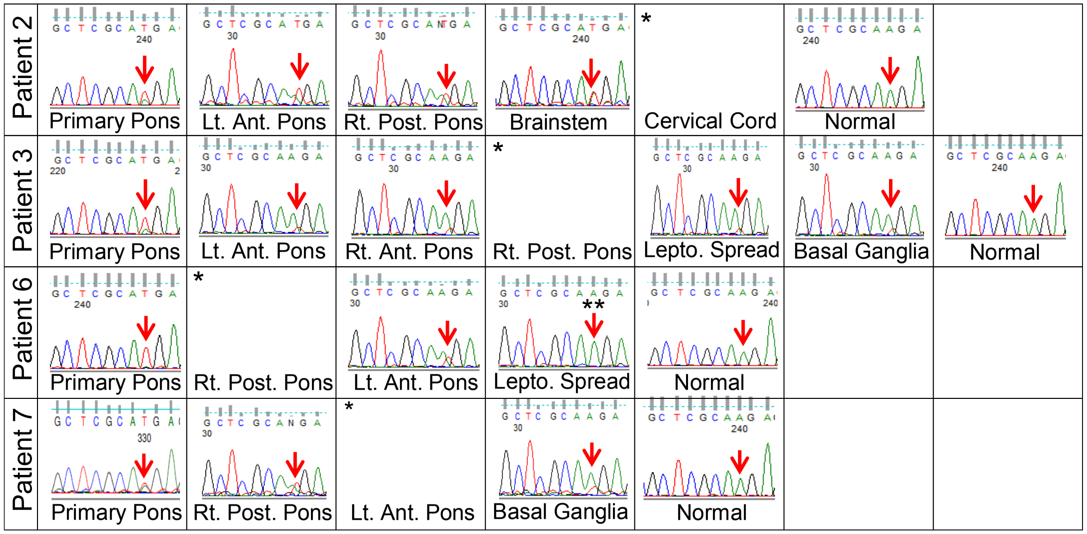

Online Resource 5. Chromats of sanger sequencing results for a) HIST1H3B and b)H3F3A K27M

Article Title: Spatial genomic heterogeneity in diffuse intrinsic pontine and midline high-grade glioma: implications for diagnostic biopsy and targeted therapeutics
Journal Name: Acta Neuropathologica
Author Names: Lindsey M. Hoffman[1*], Mariko DeWire[1*], Scott Ryall[2*], Pawel Buczkowicz[2], James Leach[1], Lili Miles[1], Arun Ramani[2], Michael Brudno[2], Shiva Senthil Kumar[1], Rachid Drissi[1], Phillip Dexheimer[1], Ralph Salloum[1], Lionel Chow[1], Trent Hummel[1], Charles Stevenson[1], Qing Lu[1], Blaise Jones[1], David Witte[1], Bruce Aronow[1], Cynthia E. Hawkins[2**], Maryam Fouladi[1**]
Author Affiliations: 1 – Cincinnati Children's Hospital Medical Center, Cincinnati, Ohio; 2 – The Hospital for Sick Children, Toronto, Canada
Email of Corresponding Author: maryam.fouladi@cchmc.org
