# Supplementary material for: Spatial genomic heterogeneity in diffuse intrinsic pontine and midline high-grade glioma: implications for diagnostic biopsy and targeted therapeutics
Source: Acta Neuropathol Commun. 2016 Jan 4;4:1. doi: 10.1186/s40478-015-0269-0 (PMC4700584; doi:10.1186/s40478-015-0269-0)
Supplement: Additional file 6: Figure S2. — Immunohistochemistry validation of TP53 mutants. (PDF 216 kb) [file 40478_2015_269_MOESM6_ESM.pdf]

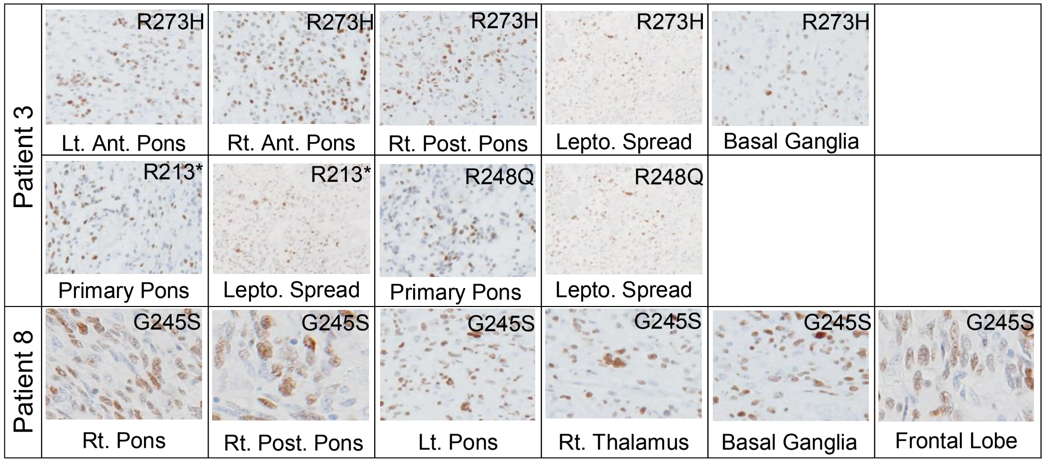


Online Resource 6. Immunohistochemistry validation of TP53 mutants

Article Title: Spatial genomic heterogeneity in diffuse intrinsic pontine and midline high-grade glioma: implications for diagnostic biopsy and targeted therapeutics

Journal Name: Acta Neuropathologica

Author Names: Lindsey M. Hoffman[1*], Mariko DeWire[1*], Scott Ryall[2*], Pawel Buczkowicz[2], James Leach[1], Lili Miles[1], Arun Ramani[2], Michael Brudno[2], Shiva Senthil Kumar[1], Rachid Drissi[1], Phillip Dexheimer[1], Ralph Salloum[1], Lionel Chow[1], Trent Hummel[1], Charles Stevenson[1], Qing Lu[1], Blaise Jones[1], David Witte[1], Bruce Aronow[1], Cynthia E. Hawkins[2**], Maryam Fouladi[1**]
Author Affiliations: 1 – Cincinnati Children's Hospital Medical Center, Cincinnati, Ohio; 2 – The Hospital for Sick Children, Toronto, Canada
Email of Corresponding Author: maryam.fouladi@cchmc.org
